# Re-TEVAR in treatment of stent graft-induced new entry between two non-overlapping stent-grafts: a case report

**DOI:** 10.1186/s13019-020-01359-w

**Published:** 2020-10-15

**Authors:** Yiwei He, Shoujun Tang, Yongheng Zhang, Jianping Liu, Haining Zhou

**Affiliations:** 1 Zunyi Medical University, Zunyi, 653000 China; 2 Department of Cardiothoracic Surgery, Suining Central Hospital, No. 127, Desheng Road, Chuanshan District, Suining, 629000 China

**Keywords:** Stent graft-induced new entry, Thoracic endovascular aortic repair, Type B aortic dissection

## Abstract

**Background:**

Progress of the aortic disease after the stent graft treatment of aortic dissection implicates the potential risks of stent graft-induced new entry (SINE). Although rarely reported, it should be vigilant in patients who might incur serious complication in early period after the thoracic endovascular aortic repair (TEVAR). Thus, the development of aortic disease-specific stent grafts would assist in achieving positive patient outcomes when suffering SINE. However, it is an extremely rare for SINE between two non-overlapping stent-grafts.

**Case presentation:**

We here reported a 59-year-old male patient with sudden onset of chest pain for 4 h. Multi-detector computed tomography (MDCT) revealed a huge SINE formed between two non-overlapping stent-grafts. The re-TEVAR surgery was performed and the patient experienced a good recovery.

**Conclusion:**

The SINE between two non-overlapping stent-grafts treated by re-TEVAR operation was alternative and feasible. The short-term and medium-term follow-up results were satisfactory.

## Introduction

Thoracic endovascular aortic repair (TEVAR) was first reported in 1999 and its first-generation equipment was applied to endovascular treatment of thoracic aortic aneurysm, and subsequently widely applied to Stanford B aortic dissection (TBAD) [1]. Stent graft-Induced New Entry (SINE) appears to be an uncommon iatrogenic phenomenon typically occurring as a late complication of the endovascular treatment of aortic dissections, specifically in TBAD. This complication was defined as a new tear caused by the stent graft, excluding natural disease progression or iatrogenic injury, which can lead to high mortality rates. To our knowledge, it is an extremely rare case for SINE between two non-overlapping stent-grafts. In this case report, the patient with SINE between two non-overlapping stent-grafts were successfully treated by a re-TEVAR operation.

## Case presentation

A 59-year-old male patient was admitted to our hospital with sudden onset of chest pain for 4 h. The patient had a longstanding history of controlled hypertension.

**Fig. 1:**
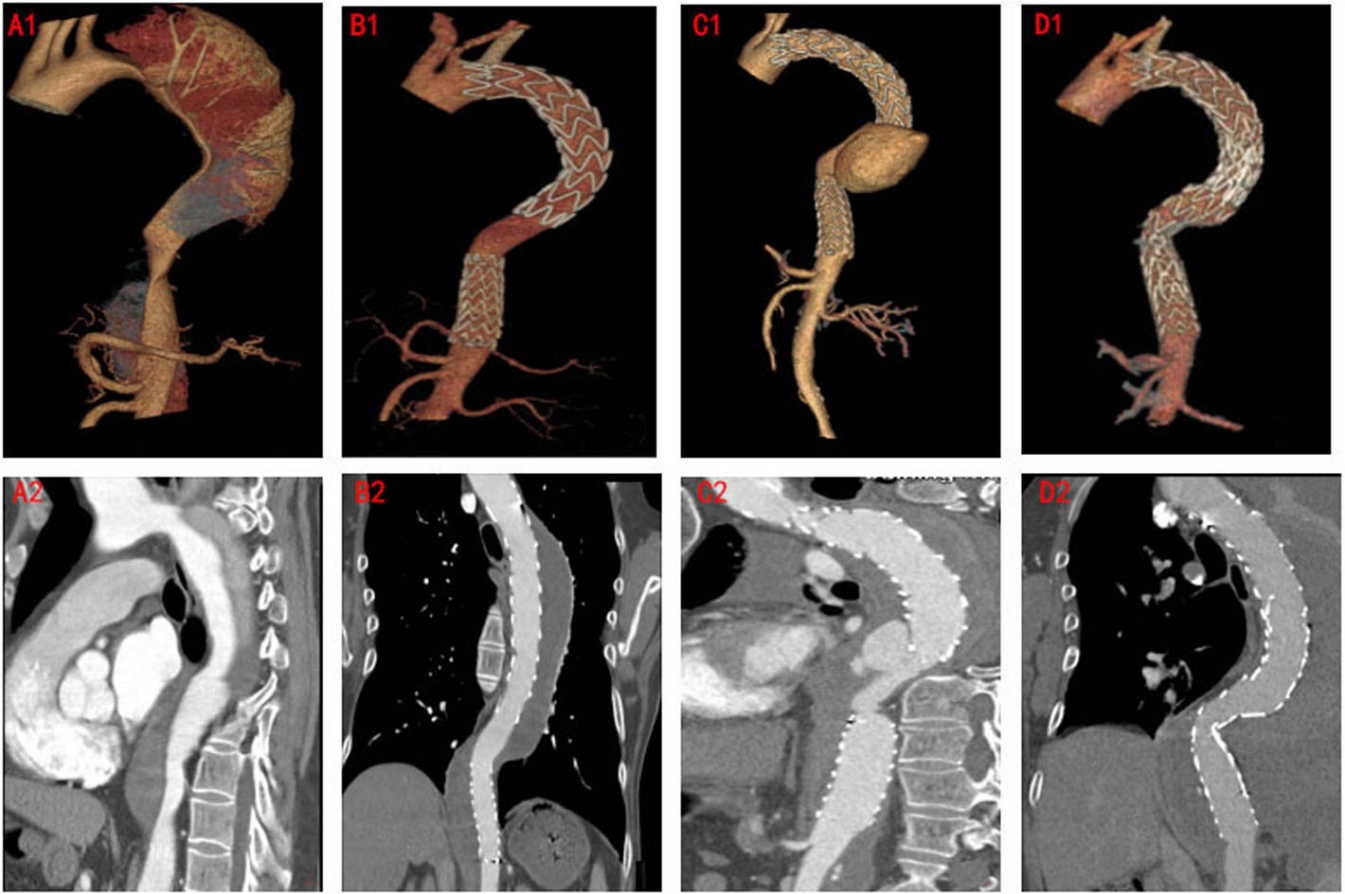
**a1**, **a2** The MDCT showed proximal and distal breaks before the primary TEVAR. **b1, b2** The area of non-overlapping two stent-grafts was normal at 4 months after primary TEVAR. **c1, c2** 3 years after the primary TEVAR, a SINE between two non-overlapping stent-grafts was formed. **d1, d2** The SINE between two non-overlapping stent-grafts was disappeared by re-TEVAR

Three years ago, the patient presented himself to a local hospital with sudden onset of chest and abdominal pain. Multi-detector computed tomography (MDCT) revealed aortic arch dissection combined with distal dissection of descending aorta (Fig. 1a1, a2), of the case who had undergone the primary TEVAR assisted by Chimney technique with two non-overlapping stent-grafts surgery at that time. The proximal breaks were covered with a stent-graft (Medtronic VA 3030C200TE) assisted by Chimney technique (Fluency 10 mm*40 mm). The second stent-graft (Medtronic ENE2828C80EE) was implanted above the abdominal trunk artery to cover the distal breaks. In order to cover the breaks as much as possible and reduce the risk of paraplegia due to the continuous coverage, the two stent-grafts were non-overlapped, and the distance was about 30 mm. At 4 months after the primary TEVAR, MDCT did not show any significant change in the repaired aorta (Fig. 1b1, b2). However, 3 years later, MDCT showed a SINE between two non-overlapping stent-grafts had develop into a huge aneurysm originating from the distal end of the first stent-graft to the proximal end of the second stent-graft (Fig. 1c1, c2). Considering the possibility of a fatal aneurysm rupture at any time, emergent re-TEVAR was performed, and the third stent-graft (XianjianXJZDF36180) was implanted to prevention of aneurysm rupture, instead of open surgery. The third stent-graft was carefully implanted according to the overlapping area by re-TEVAR, and overlapped the distal end of the first stent-graft more than 70 mm, and the proximal end of the second stent more than 60 mm. MDCT re-examination showed the SINE between the primary no-overlapping stent-grafts disappeared and the reconstruction of blood vessels was smooth within 2 years of clinical follow-up checkup (Fig. 1d1, d2). The patient experienced a good recovery after surgery without stent migration, collapse, stroke, and spinal cord ischaemia.

## Discussion and conclusion

SINE is an uncommon late complication of the endovascular treatment of aortic dissections with high mortality rates [2]. It can occur either at the proximal or distal end of the stent [2, 3]. SINE, which was first reported by Kato et al. [4] in 2001 as an aneurysmal degeneration of the aorta after TEVAR for acute aortic dissection, is one of the serious and specific complications. Risk factors of SINE may include the oversizing of the stent graft, the radial force of an oversized stent graft or an already diseased intima [4]. By generalizing the past researches, Wadi concluded that the etiology of SINE was likely related to the radial force of an oversized stent graft, the oversizing of the stent graft, intrinsic intimal weakness, a false lumen remodeling, or natural progression of the aortic disease [5]. Thus, the reintervention for SINE can excavate new entry around the aorta and avoid the aortic rupture. The treatment of proximal and distal SINE had been reported in previous clinical cases [6–8]. Some researchers had suggested that the risk of paraplegia could be significantly reduced by preserving as many intercostal arteries as possible [9, 10]. It was worth to be considered to avoid potential complications induced by paraplegia due to the long frame coverage area [9–11]. Meanwhile, the vascular true-lumen could be open up as much as possible. Given above concerns, these two stent-grafts were non-overlapped and the intercostal artery was preserved in the T7-L2 segment of the descending aorta.

To our knowledge, it is the first-reported case of the two non-overlapping stent-grafts of SINE treated by re-TEVAR. Frankly, we would not deny that the original TEAVR might be a failed operation, but the results are satisfactory after re-TEVAR. The SINE between two non-overlapping stent-grafts was treated successfully and the descending aorta was well recovered by re-TEVAR. No paraplegia was observed after re-TEVAR. It will be an ideal option for patients to suffer from less risk of cardiopulmonary bypass and deep hypothermia circulatory arrest.

As one of the complications after TEVAR, SINE may be more dangerous than TBAD itself, the SINE between two non-overlapping stent-grafts treated by re-TEVAR operation is alternative and feasible. The short-term and medium-term follow-up results are satisfactory.

## Data Availability

All data analyzed during this study are included in this published article.

## Abbreviations

TEVAR: Thoracic endovascular aortic repair
TBAD: Stanford B aortic dissection
SINE: Stent graft-Induced New Entry
MDCT: Multi-detector computed tomography
